# 623 Multi-Modal Analgesia for > 10% TBSA Pediatric Burns

**DOI:** 10.1093/jbcr/irac012.251

**Published:** 2022-03-23

**Authors:** Angela Wratney, Erin Beitz, Tamara Roberts, Margaret Anderson

**Affiliations:** SUNY Upstate Medical University, Syracuse, New York; SUNY Upstate Medical University, Syracuse, New York; Upstate, Blossvale, New York; SUNY Upstate Medical University, Syracuse, New York

## Abstract

**Introduction:**

A review of literature revealed no analgesia or opioid sparing treatment guidelines for > 10% TBSA pediatric burns. Clinicians may benefit from an analgesia support guidance tool for the management of critically ill PICU and non-PICU inpatient pediatric burn patients with > 10% TBSA.

**Methods:**

A multi-disciplinary team of experts created a pediatric analgesia support guidance tool for clinicians managing non-procedural pain in critically ill pediatric burn patients in the PICU and non-PICU inpatient setting. Agent selection was at the discretion of the clinician based on FLACC scoring. We performed a retrospective chart review, Jan 2019 to Jun 2021, of pediatric patients age 1 month to 14 years of age admitted to the Pediatric Burn Trauma service with > 10%TBSA. Analgesia was categorized as Yes/No for acetaminophen, ibuprofen, opioids (morphine, fentanyl, oxycodone, hydromorphone), gabapentin, alpha 2-agonists (dexmedetomidine, clonidine).

**Results:**

16 pediatric burn patients age 2 months to 14 years. 75% male, 4 (25%) PICU, 3 (19%) mechanically ventilated. Average LOS 7.19 days, range 1 to 25 days. 9 (56%) received standing acetaminophen, 1(6%) ibuprofen, 12 (75%) opioids, 4 (25%) gabapentin, 3 (19%) alpha-2 agonists.

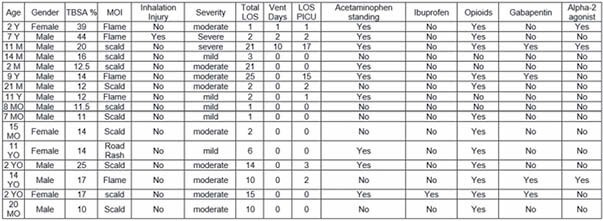

**Legend:**

Y, years; M, months; TBSA Total Body Surface Area; MOI mechanism of injury; LOS length of stay; vent ventilator; PICU pediatric intensive care unit;

**Conclusions:**

We found 75% of our pediatric patients received opioids, and only 56% received standing acetaminophen. 7 patients (43.8%) received either gabapentin and/or alpha-2 agonists. Our pediatric analgesia support guidance tool influenced clinician pain management practice for the pediatric burn patient. With continued use, we will determine if this multimodal approach can reduce an over reliance on opioid analgesia regimens.

